# Propofol attenuates lung ischemia/reperfusion injury though the involvement of the MALAT1/microRNA-144/GSK3β axis

**DOI:** 10.1186/s10020-021-00332-0

**Published:** 2021-07-15

**Authors:** Jian-Ping Zhang, Wei-Jing Zhang, Miao Yang, Hua Fang

**Affiliations:** 1 Department of Anesthesiology, Guizhou Provincial People’s Hospital, No. 83, East Zhongshan Road, Guiyang, Guizhou 550002 People’s Republic of China; 2 Department of Anesthesiology, Guizhou University People’s Hospital, No. 83, East Zhongshan Road, Guiyang, Guizhou 550025 People’s Republic of China; 3 Laboratory of Anesthesiology & Perioperative Medicine, Guizhou University School of Medicine, Guiyang, 550025 People’s Republic of China

**Keywords:** Propofol, Lung ischemia/reperfusion injury, Metastasis-associated lung adenocarcinoma transcript, Microrna-144, Glycogen synthase kinase-3β, Autophagy

## Abstract

**Background:**

Propofol, an intravenous anesthetic, was proven to protect against lung ischemia/reperfusion (I/R) injury. However, the detailed mechanism of Propofol in lung I/R injury is still elusive. This study was designed to explore the therapeutic effects of Propofol, both in vivo and in vitro*,* on lung I/R injury and the underlying mechanisms related to metastasis-associated lung adenocarcinoma transcript 1 (MALAT1)/microRNA-144 (miR-144)/glycogen synthase kinase-3β (GSK3β).

**Methods:**

C57BL/6 mice were used to establish a lung I/R injury model while pulmonary microvascular endothelial cells (PMVECs) were constructed as hypoxia/reperfusion (H/R) cellular model, both of which were performed with Propofol treatment. Gain- or loss-of-function approaches were subsequently employed, followed by observation of cell apoptosis in lung tissues and evaluation of proliferative and apoptotic capabilities in H/R cells. Meanwhile, the inflammatory factors, autophagosomes, and autophagy-related proteins were measured.

**Results:**

Our experimental data revealed that Propofol treatment could decrease the elevated expression of MALAT1 following I/R injury or H/R induction, indicating its protection against lung I/R injury. Additionally, overexpressing MALAT1 or GSK3β promoted the activation of autophagosomes, proinflammatory factor release, and cell apoptosis, suggesting that overexpressing MALAT1 or GSK3β may reverse the protective effects of Propofol against lung I/R injury. MALAT1 was identified to negatively regulate miR-144 to upregulate the GSK3β expression.

**Conclusion:**

Overall, our study demonstrated that Propofol played a protective role in lung I/R injury by suppressing autophagy and decreasing release of inflammatory factors, with the possible involvement of the MALAT1/miR-144/GSK3β axis.

**Supplementary Information:**

The online version contains supplementary material available at 10.1186/s10020-021-00332-0.

## Introduction

Lung ischemia/reperfusion (I/R) injury is a primary cause of dysfunction in pulmonary grafts, which plays a significant role in lung disease treatment (Wu et al. 2015). I/R injury is characterized by damage of cells in certain organ systems under hypoxic conditions, accompanied by a sudden restoration of oxygenation to tissues (Mendes-Braz et al. 2012). In lung diseases, the destruction of vascular endothelial cells and alveolar epithelial cells caused by inflammation may affect the normal respiratory function of the lungs, thus reducing cellular oxygen partial pressure of the lung tissues and causing the body to initiate a hypoxic response (Sharma et al. 2018). In addition, autophagy participates in cancers and other dysfunctions (Saha et al. 2018). Of note, it has been demonstrated that autophagy is implicated in the pathophysiological process of lung I/R injury (Zhang et al. 2013). However, the specific role of autophagy in lung I/R injury has still not been adequately studied.

In recent years, Propofol (2,6-di-isopropyl phenol), an intravenous anesthetic, has been found to exert protective effects on lung injury induced by intestinal I/R (Li et al. 2019). Furthermore, Propofol could lessen acute lung injury induced by renal I/R involved with autophagy (Liu et al. 2020). The detailed regulatory mechanism of Propofol in lung I/R injury remains largely unclear, which warrants further investigations. Long non-coding RNAs (lncRNAs) are a group of RNAs not encoding proteins associated with I/R injury (Wolska et al. 2020). LncRNA metastasis-associated lung adenocarcinoma transcript 1 (MALAT1), located at chromosome 11q13, has been reported to be a key participant in the Propofol-mediated mechanism in gastric cancer (Zhang et al. 2020). Silencing MALAT1 also inhibits inflammation in lung I/R injury (Wei et al. 2019). In addition, MALAT1 has been found to regulate the expression of microRNA (miR)-144 in myocardial I/R injury to mediate the inflammatory response (Chen et al. 2019). miR-144 serves as a therapeutic target to alleviate cardiac I/R injury (Lusha et al. 2019). However, the role of miR-144 in lung I/R injury still needs further exploration. Inactivated glycogen synthase kinase-3β (GSK3β) is has been proven to protect myocardial cells from I/R injury (Wang et al. 2020), yet it is not known how GSK3β affects lung I/R injury.

Based on the above research, we established a mouse model of I/R injury and a hypoxia/reperfusion (H/R) cell model to validate our hypothesis that Propofol exerts protective effects on I/R-induced lung injury through the MALAT1/miR-144/GSK3β axis.

## Materials and methods

### Ethical statement

The animal experiment was conducted with the approval from the Animal Ethics Committee from Guizhou Provincial People’s Hospital.

### Establishment Of lung I/R injury mouse model

C57BL/6 J mice (aged 6—8 weeks, weighing 20—26 g) were purchased from the Animal Experimental Center, Chinese Academy of Sciences and anesthetized by peritoneal injection of 3% sodium pentobarbital (50 mg/kg). During the operation, the mice were placed on a heating pad to maintain constant temperature. Mice in the sham group (n = 8) were subjected to left thoracotomy without occlusion of pulmonary hilum with mechanical ventilation maintained for 3 h. Mice in the I/R group (n = 8) were subjected to left thoracotomy followed by occlusion of left pulmonary hilum with a non-invasive vascular clamp for 1 h, and reperfusion was performed on the pulmonary hilum, followed by ventilation for 2 h. Mice in the I/R + Propofol group (n = 8) were injected with Propofol 36 mg/(kg*h) into the femoral vein 30 min before occlusion of the pulmonary hilum, and the other operations were conducted identically with those in the I/R group (Additional file 1: Fig. S1).

Adenovirus-based (1 × 10^11^ PFU, 50 μL) MALAT1, negative control (NC), or GSK3β overexpressed vector (oe-MALAT1, oe-NC, or oe-GSK3β) was provided by Geneland Biotechnology (Shanghai, China), which was injected through the caudal vein into mice 48 h before modeling. Next, the left hilum was left exposed for 1 h followed by reperfusion for 2 h. Mice were grouped into the I/R + oe-NC group ( n = 8), the I/R + oe-MALAT1 group (n = 8), the I/R + oe-GSK3β group (n = 8), the I/R + oe-NC + Propofol group (n = 8), the I/R + oe-MALAT1 + Propofol group (n = 8), the I/R + oe-GSK3β + Propofol group (n = 8), the I/R + oe-MALAT1 + dimethylsulfoxide (DMSO) + Propofol group (n = 8), and the I/R + oe-MALAT1 + GSK inhibitor SB216763 + Propofol group (n = 8).

At the end of the perfusion period, blood-gas values were recorded from 0.4 mL of heparinized blood drawn from the left pulmonary vein, and mice were later euthanized by intravenous injection of excess sodium pentobarbital. The left lung was immediately excised and lung tissues were cut into several parts, some of which were preserved in 10% formaldehyde, heated at 70 °C for 72 h, and fixed in 2% glutaraldehyde and oligoformaldehyde. Meanwhile, the remaining parts were frozen in liquid nitrogen for later use. Propofol is provided by Libang Sciences (Xi’an, China).

### Arterial oxygen tension (PaO_2_) level in mice

At the end of pulmonary reperfusion, 1 mL empty syringe was filled with heparin. Approximately 0.4 mL of arterial oxygenated blood was extracted from the left ventricle of mice. The PaO_2_ level was measured and recorded by Nova Biomedical automatic blood gas analyzer immediately to evaluate the oxygenation function of pulmonary tissues (Wei et al. 2019).

### Wet/Dry (W/D) ratios in mouse lung tissues

The fresh lung tissues to be tested were washed, dried with filter paper, placed on a balance, and weighed, which was recorded as W. The same tissues were placed in a drying oven at a constant temperature of 70 °C for 72 h until the tissues were completely dehydrated. The dry weight was weighed and recorded as D. The ratio of W/D was calculated to evaluate the degree of edema in injured lung tissues.

### Hematoxylin & eosin (H&E) staining

The left lung tissues of mice in different groups were collected and fixed in 10% formaldehyde to prepare paraffin sections. After sections were left to dry at room temperature, they were washed with 1 × phosphate buffer saline (PBS) for 3 s, stained with hematoxylin for 60 s, washed with 1% hydrochloric alcohol differentiation liquid for 3 s, stained with eosin for 3 min, and hydrated by gradient ethanol (70%, 80%, 95%) and anhydrous ethanol 5 min each time. Sections were then dewaxed by xylene and finally mounted with neutral resins. Pathological changes of lung injury were observed under a light microscope (BX63, Olympus, Japan).

### Scoring of lung tissue injury

A total of 300 alveoli were counted on each slide, and lung injury index was scored according to the established criteria (Matute-Bello et al. 2001) with the following formula: injury score = [(alveolar bleeding point/number of visual field) + 2 × (alveolar infiltration point/number of visual field) + 3 × (fibrin point/number of visual field) + (alveolar septal congestion/number of visual field)]/total alveolar count.

### Terminal deoxynucleotidyl transferase-mediated deoxyuridine triphosphate nick end-labeling (TUNEL) staining

Cell apoptosis in lung tissues was determined by following the instructions of a TUNEL apoptosis detection kit (Millipore, Billerica, MA, USA). Paraffin-embedded lung tissue sections from mice were dewaxed in xylene for 5—10 min, dehydrated with gradient ethanol (90%, 70%), washed with distilled water for 2 min, and reacted with 20 μg/mL DNase-free protease K (ST532, Beyotime Biotechnology, Shanghai, China) at 20—37 °C for 15—30 min. The sections were then incubated in 3% hydrogen peroxide solution prepared with PBS for 20 min at room temperature, biotin-labeled solution for 60 min at 37 °C, and labeling reaction termination solution for 10 min at room temperature. Afterwards, the sections were incubated with 50 μL of streptavidin-horseradish peroxide (HRP) working solution for 30 min at room temperature and developed with 0.2—0.5 mL diaminobezidin (DAB) chromogenic solution for 5—30 min at room temperature. Subsequently, the sections were mounted, observed, and photographed under an inverted microscope, with ten fields of view randomly selected in each group. Positive cells and total cells were counted. Cells with brownish-yellow nuclei were apoptotic-positive cells, while cells with blue nuclei were normal cells. The apoptotic rate was calculated with the following formulae: the number of brown yellow cells/the number of blue cells × 100%.

### Isolation of pulmonary microvascular endothelial cells (PMVECs)

Mice were anesthetized and thoracotomized for drainage. Subpleural lung tissues were isolated and the tissues at a depth of 1 mm at the lung margin were cut into 1—3 mm^3^ pieces, which were immersed in Roswell Park Memorial Institute-1640 (RPMI-1640) culture medium (11,875,101, Gibco, Green Land, USA) containing 10% fetal bovine serum (FBS; 100,099,141, Gibco) and cultured in an incubator at 37 °C with 5% CO_2_. The medium was renewed daily to remove blood cells. Tissue blocks were cultured in a culture dish for 60 h. Next, the tissues were gently removed and the cells were cultured until 90% confluency. The cells were then detached and passaged with 0.25% trypsin without ethylene diamine tetraacetic acid. Cells at passage 3 were used for the subsequent experiments. Endothelial cell marker CD31 staining (rabbit anti-CD31, ab182981, 1: 1000, Abcam, Cambridge, UK) was performed to confirm the cell purity.

### Cell culture and transfection

Primary PMVECs were cultured in RPMI-1640 medium (11875101, Gibco) containing 10% FBS (100099141, Gibco), while 293 T cells (Paitong Biotechnology, Shanghai, China) were cultured in Dulbecco’s modified eagle medium (DMEM) added with 10% FBS, 1% penicillin–streptomycin (15070063, Gibco) and 1% glutamine (25030081, Gibco). Cell culture was performed in a cell incubator at 37 °C with 5% CO_2_.

Silenced and overexpressed lentiviruses were packaged with core plasmids (pLKO.1) and auxiliary plasmids (psPAX2, pMD2.G) inserted with target gene silencing sequence, and core plasmids (pHAGE-CMV-MCS-IzsGreen) and auxiliary plasmids (psPAX2, pMD2.G) inserted into the target gene cDNA sequence, respectively. Lentiviruses were purchased from Sangon Biotech (Shanghai, China). Primer sequences and plasmids were also constructed by Sangon Biotech. The vector encapsulating the virus and the target was co-transfected into 293 T cells using Lipo2000 (11668-019, Invitrogen, NY, USA). The supernatant was collected after 48 h of cell culture. Viral particles were filtered from the supernatant and centrifuged, followed by detection of viral titers. The virus in the logarithmic growth phase was collected and divided based on transfectants into short hairpin RNA (shRNA) targeting NC, MALAT1-1, and MALAT1-2 (sh-NC, sh-MALAT-1, sh-MALAT-2), vector overexpressing NC, MALAT1, and GSK3β (oe-NC, oe-MALAT1, oe-GSK3β), plasmids of mimic NC, miR-144 mimic, inhibitor NC, and miR-144 inhibitor. Plasmids of the mimic and inhibitor were purchased from GenePharma (Shanghai, China). Cells in the logarithmic phase were trypsinized and triturated to 5 × 10^4^ cells/mL cell suspension, which was seeded in 6-well plates with a density of 2 mL per well and left to culture overnight at 37 °C. After 48 h of infection, expression of related genes in cells of each group was detected by reverse transcription quantitative polymerase chain reaction (RT-qPCR) and repeated three times. Silencing lentivirus transfection and silencing sequences are shown in Additional file 2: Table S1. Lentivirus-treated cell lines were screened and constructed into stable cell lines.

### Establishment of H/R PMVEC model

When cell confluency reached approximately 80%, normal cell culture medium was renewed with serum-free and glucose-free DMEM. The cells were placed in a hypoxic incubator containing 95% N_2_ and 5% CO_2_ for 12 h under hypoxic conditions. Next, the culture medium was replaced with DMEM (10,569,044, Gibco) containing glucose, 1% penicillin–streptomycin, 10% FBS and 4 mM L-glutamine for 4 h to construct the H/R cell model. The cells were collected for subsequent experiments. For the H/R + Propofol cell model, 100 μm/L Propofol (Libang Sciences) was added prior to the establishment of H/R cell model (Additional file 3: Fig. S2).

### Cell counting kit (CCK) 8 assay

Cell viability after Propofol treatment was assessed using a CCK8 kit (K1018, APExBIO, Huston, USA). Cells were coated on 96-well plates with 1 × 10^4^ cells per well (100 μL/well) and treated with H/R and Propofol under hypoxic conditions. Propofol was repeatedly administered in 6 wells at a concentration 100 μm/L, and non-H/R cells were treated with Propofol for 16 h. After treatment, the cells were washed twice with PBS and incubated with 10 μL CCK8 solution at 37 °C for 2 h. The absorbance value at 450 nm was determined by a microplate reader, and cell viability (%) was calculated using the following formulae, [(As-Ab)/(Ac-Ab)] × 100%, in which “As” refers to the absorbance value of supernatant from exposed or sham exposure dishes; “Ac” refers to the absorbance value of pore containing supernatant in normal control; “Ab” refers to the absorbance value of cultured pore containing 10% CCK8 solution.

### Treatment of GSK3β inhibitor

A total of 10 μM GSK3β inhibitor SB216763 (10 mM inhibitor mother liquor dissolved in DMSO (S1075, Selleck Chemicals, Houston, TX, USA) was added to the cell culture medium and treated for 12 h. Cells were induced by H/R. As for the in vivo experiment, mice were injected with SB216763 at a dose of 20 mg/kg through the femoral vein 30 min before I/R, while the mice in the control group were injected with the same volume of DMSO.

### Immunohistochemical staining

The lung tissue sections of mice were heated at 60 °C for 20 min, soaked in xylene solution and renewed xylene solution for 15 min each. Sections were dehydrated by gradient ethanol (100%, 95%, 90%, 85%, and 80%) and washed several times with double distilled water. Following that, each section was dripped with 3% H_2_O_2_ and soaked for 10 min at room temperature to block endogenous peroxidase. Next, the sections were added with citric acid buffer, boiled in a microwave oven for 3 min, dripped with antigen repair solution at room temperature for 10 min, added with normal goat serum blocking solution (Sangon Biotech) at room temperature for 20 min and incubated with primary antibodies of rabbit anti-GSK3β (#12,456, 1: 200, Cell Signaling Technology, Danvers, MA, USA) overnight in complete darkness. On the next day, the sections were incubated with goat anti-rabbit immunoglobulin G (IgG; ab6721, 1: 1000, Abcam) secondary antibodies for 30 min and with streptAvidin–Biotin Complex (Vector company, Olean, NY, USA) in an incubator at 37 °C for 30 min. Color was developed by DAB developing kit (P0203, Beyotime Biotechnology) for 6 min. Sections were then stained with hematoxylin for 30 s, and dehydrated by 70%, 80%, 90%, and 95% ethanol and absolute ethanol, 2 min each. Finally, sections were cleared with xylene for 5 min, mounted by neutral resin, and observed under an upright microscope (BX63, Olympus, Japan).

### Flow cytometry

Cell apoptosis was detected by Annexin V-fluorescein isothiocyanate (FITC)/propidium iodide (PI) apoptosis detection kit (C1062M, Beyotime Biotechnology). A total of 1 × 10^6^c ells/mL were washed twice with cold PBS and then gently resuspended with 195 μL Annexin V-FITC binding solution. Cells were then incubated with 5 μL Annexin V-FITC and 10 μL PI at room temperature in the dark for 15 min. Cell apoptosis was quantified by a flow cytometer (FACSVerse/Calibur/AriaIISORP, BD Biosciences, San Jose, CA, USA).

### Dual-luciferase reporter gene assay

The possible binding sites of miR-144 to GSK3β were predicted online using JASPER tool. The 3'untranslated region (UTR) dual-luciferase reporter gene vector of GSK3β and the mutant plasmids with mutations in the binding site of miR-144 were constructed, as PmirGLO-GSK3β-wild type (WT) and PmirGLO-GSK3β-mutant type (MUT). The reporter plasmids of miR-144 mimic mimic NC were co-transfected into 293 T cells, which were lysed 48 h after transfection. Cells were centrifuged at 12,000 rpm for 1 min and the supernatant was collected. Luciferase activity was then detected using the Dual-Luciferase® Reporter Assay System (E1910, Promega, Madison, WI, USA). Each cell sample was added with 100 μL firefly luciferase working solution to determine the Firefly luciferase, while the addition of 100 μL renilla luciferase working solution was performed to measure the Renilla luciferase. The luciferase activity was expressed as the ratio of LU1/RLU2, in which LU1 refers to firefly luciferase reaction intensity, and RLU2 refers to the internal reference renilla luciferase reaction intensity.

### RNA pull-down

PMVECs were transfected with 50 nM biotin-labeled WT-bio-miRNA-144 and MUT-bio-miRNA-144 (GeneCreate, Wuhan, China). After 48 h of transfection, cells were collected and washed with PBS. Cells were incubated in specific lysis buffer (Ambion, Austin, TX, USA) for 10 min. The lysate was incubated with M-280 streptavidin magnetic beads (S3762, Sigma, St. Louis, MO, USA) pre-coated with RNase-free bovine serum albumin (BSA) and yeast tRNA (TRNABAK-RO, Sigma), and incubated at 4 °C for 3 h. Cells were washed twice with precooled lysis buffer, three times with low salt buffer, and once with high salt buffer. The bound RNA was purified by Trizol and the enrichment of MALAT1 was detected by RT-qPCR.

### Western blot analysis

Tissues and cells were lysed by the addition of radio-immunoprecipitation assay lysis buffer containing phenylmethanesulfonyl fluoride (P0013B, Beyotime Biotechnology). Proteins were extracted by following the instructions of a kit (P0028, Beyotime Biotechnology). The total protein concentration of each sample was determined by a bicinchoninic acid kit (P0011, Beyotime Biotechnology). Sodium dodecyl sulfate (8–12%) gel was prepared based on the target protein bands, and the protein samples were electrophoresed for separation. Proteins on the gel were transferred to a polyvinylidene fluoride membrane (1620177, BIO-RAD, Hercules, CA, USA), which was blocked by 5% skimmed milk or 5% BSA for 1 h at room temperature. Rabbit anti-glyceraldehye phosphate dehydrogenase (GAPDH; #2118, 1: 5000, Cell Signaling Technology), rabbit anti-GSK3β (#12456, 1: 5000, Cell Signaling Technolog), rabbit anti-light chain 3B (LC3B) (ab51520, 1: 3000, Abcam), and rabbit anti-Beclin1 (ab207612, 1: 2000, Abcam) were added and incubated with the membrane overnight at 4 °C. Afterwards, the membrane was rinsed with 1 × Tris-buffered saline Tween three times at room temperature for 5 min each. Goat anti-rabbit IgG (ab6721, 1: 5000, Abcam) secondary antibodies labeled by HRP were dripped in the membrane and incubated for 1 h at room temperature. The membrane was immersed in an enhanced chemiluminescence reaction solution (1705062, Bio-Rad) at room temperature for 1 min. After the liquid was aspirated, the membrane was covered with a preservative film, and the protein bands were exposed on an Image Quant LAS 4000C gel imager (GE Healthcare, Princeton, NJ, USA). GAPDH was used as the internal reference for total cell protein and cytoplasmic protein content, and the ratio of gray value of target band to internal reference band was used as the relative protein expression.

### RT-qPCR

The total RNA content of cells was extracted with Trizol (16096020, Thermo Fisher Scientific, Waltham, MA, USA) and reversely transcribed into complementary DNA (cDNA) using PrimeScript RT kit (Takara Biotechnology, Dalian, China) and PrimeScript miRNA RT kit (Takara Biotechnology). RT-qPCR was assayed with a RT-qPCR kit (Q511-02, Vazyme Biotech, Nanjing, China) by following the instructions provided. With 2 μL cDNA as templates, the forward and reverse primers (each 0.2 μL) were mixed with 10 μL RT-qPCR and supplemented with RNAase-free water to 20 μL. PCR amplification was carried out with Bio-rad real-time qPCR instrument (CFX96, Bio-Rad). MALAT1 was normalized to GAPDH level, while miR-144 was normalized to U6. The primer sequences were designed and provided by Sangon Biotech. The primer sequences are shown in Additional file 4: Table S2. The gene expression was measured using the 2-^ΔΔCt^ method with the formula as follows: ΔΔC_t_ = ΔC_t__experimental group_−ΔC_t__control group_, where ΔC_t_ = C_t__(target gene)_−C_t__(internal reference)_.

### Immunofluorescence against LC3

Cells were fixed with 4% paraformaldehyde for 20 min, washed three times with PBS for 5 min each time, and then incubated with Triton X-100 (Sigma) for 15 min to increase membrane permeability. After cells were blocked with BSA for 1 h, they were left to incubate overnight with rabbit antibody anti-LC3B (ab51520, 1: 2000, Abcam) at 4 °C, and then with Alexa Fluor 488-conjugated donkey anti-rabbit IgG (A21206, 1: 500, Thermo Fisher Scientific, USA) for 1 h in the dark at room temperature. Nuclei were left to stain with 4',6-diamidino-2-phenylindole in the dark for 15 min. Slides were dripped with anti-fluorescence attenuation sealing agent, and cells were observed under a confocal laser microscope (Leica TCS SP5II STED, Germany). At least 200 cells were counted in each slide and the percentage of LC3-positive cells (autophagosomes) was calculated.

### Bioinformatics analysis

I/R injury-related lncRNAs were obtained through MNDR V3.0 database (http://www.rna-society.org/mndr/) using the keyword "Ischemia/reperfusion injury". Species conservation of hmu-lncRNAs in humans, rats and mice was retrieved through UCSC Genome Browser. Downstream regulatory miRNAs of lncRNAs were predicted through the following bioinformatics prediction tools: StarBase, RNAInter and LncBase v2.0. The candidate miRNA was selected by the intersection of the predictions results using the jvenn online tool. Through the bioinformatics prediction tools StarBase, mirDIP, TargetScan and microT, the downstream mRNAs of the miRNAs were predicted, while the top 500 genes related to I/R injury ranked by Score were predicted through the GeneCards database (https://www.genecards.org/). To predict the intersection of miRNA target genes and genes related to I/R injury, a jvenn tool was further used. Related factors were screened using the GeneMANIA tool to analyze the co-expression relationship between candidate genes with the species defined as Mus musculus. Hub genes were selected for further studies according to the co-expression relationship score of the website.

### Statistical analysis

SPSS 21.0 (IBM, Armonk, NY, USA) statistical software was used for statistical analysis. The measurement data were expressed as the mean ± standard deviation. Normal distribution and homogenous variance tests were performed, followed by an unpaired *t-*test conducted on data between groups. One-way analysis of variance (ANOVA) was used for comparisons among multiple groups, Tukey's post-test was also used. A *p* < 0.05 indicates that the difference is statistically significant.

## Results

### Propofol restrains MALAT1 expression in I/R mouse model and H/R cell model

**Fig. 1:**
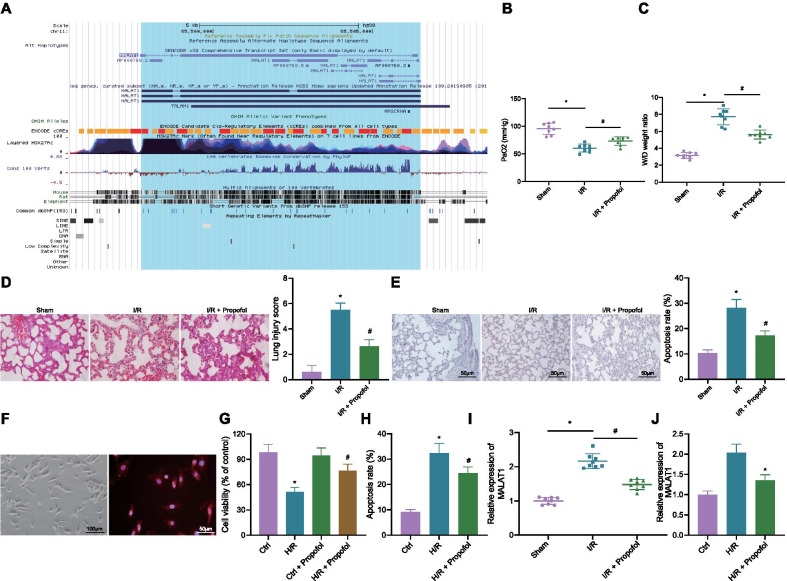
Propofol decreases the expression of MALAT1 in I/R mouse model and H/R cell model. **A** MALAT1 with high species conservation in human, rats and mice, as detected through UCSC Genome Browser (http://genome.ucsc.edu/). The gene was amplified with a magnification of 1.5 × . Heights of peak and color depth represent intensity of species conservation. **B** Measurement on PaO_2_ levels of mice (8 mice each group; *p < 0.05, compared with that of the sham group; ^#^*p* < 0.05, compared with the I/R group). **C** Measurement on W/D ratio of mice (8 mice each group; *p < 0.05, compared with that of the sham group; ^#^*p* < 0.05, compared with the I/R group). **D** Pathological changes and lung tissue injury scoring in mice, as detected by H&E staining (8 mice each group; scale bar: 25 μm, *p < 0.05, compared with that of the sham group; ^#^*p* < 0.05, compared with the I/R group). **E** Cell apoptosis in lung tissues as determined by TUNEL staining (8 mice each group; scale bar: 25 μm, *p < 0.05, compared with that of the sham group; ^#^*p* < 0.05, compared with the I/R group). **F** Morphology of PMVECs and CD14 immunofluorescent images. **G** Cell viability as assessed by CCK8 assay (*p < 0.05, compared with that of the control group; ^#^*p* < 0.05, compared with that of the control + Propofol group). **H** Cell apoptosis as assessed by flow cytometry (*p < 0.05, compared with that of the control group; ^#^*p* < 0.05, compared with the H/R group). **I** Expression of MALAT1 in lung tissues of mice as measured by RT-qPCR (8 mice each group; * *p* < 0.05, compared with that of the sham group; ^#^*p* < 0.05, compared with the I/R group). **J** Expression of MALAT1 in cells as measured by RT-qPCR (*p < 0.05, compared with that of the control group; ^#^*p* < 0.05, compared with the H/R group). Cellular experiments were repeated 3 times independently

Initially, we obtained I/R injury related lncRNAs, N1LR and MALAT1 (Additional file 5: Table S3) from the MNDR v3.0 database. MALAT1 was obtained through UCSC Genome Browser with high species conservation in humans, rats and mice (Fig. 1A). Next, the I/R mouse model was constructed by I/R surgery. The mice in the sham group showed higher PaO_2_ and lower W/D ratio than those of the mice in the I/R group. Compared with the I/R group, PaO_2_ was increased while W/D ratio was decreased in mice of the I/R + Propofol group (Fig. 1B, C). H&E and TUNEL staining results showed that compared with the sham group, the lung tissue injury score and apoptosis of PMVECs in mice of the I/R group were increased. However, when compared with the I/R group, the lung tissue injury score and apoptosis of PMVECs in mice of the I/R + Propofol group were reduced (Fig. 1D, E). These results suggested the successful construction of the I/R mouse model.

Mouse primary PMVECs were isolated and identified by morphology and CD14 immunofluorescence (Fig. 1F). Next, H/R PMVEC model was induced for in vitro study. Results of the CCK8 assay showed that the cell viability weakened distinctly after H/R treatment, and there was no significant change regarding cell viability after addition of Propofol, when compared to that of the control group. Compared with the H/R group, the cell viability strengthened in the H/R + Propofol group (Fig. 1G). Apoptosis of cells exposed to H/R was increased compared with the apoptosis rate of cells in the control group, yet the apoptosis rate of cells in the H/R + Propofol group declined compared with that of the cells in the H/R group (Fig. 1H). The above results indicated the successful induction of the H/R cell model.

The expression of MALA1 in lung tissues of mice was detected by RT-qPCR, and the results showed that the expression of MALAT1 in I/R mice was increased compared with that of sham-operated mice. The expression of MALAT1 was reduced in mice of the I/R + Propofol group compared with those of the I/R group (Fig. 1I). The expression of MALAT1 was also increased in H/R cell model compared with cells in the control group, and decreased in cells of the H/R + Propofol group compared with that of the H/R group (Fig. 1J). These results indicated that Propofol could inhibit the expression of MALAT1 in lung tissues of I/R mice as well as the H/R cell model.

### Overexpressed MALAT1 reverses protective effects of propofol on I/R mouse model and H/R cell model

**Fig. 2:**
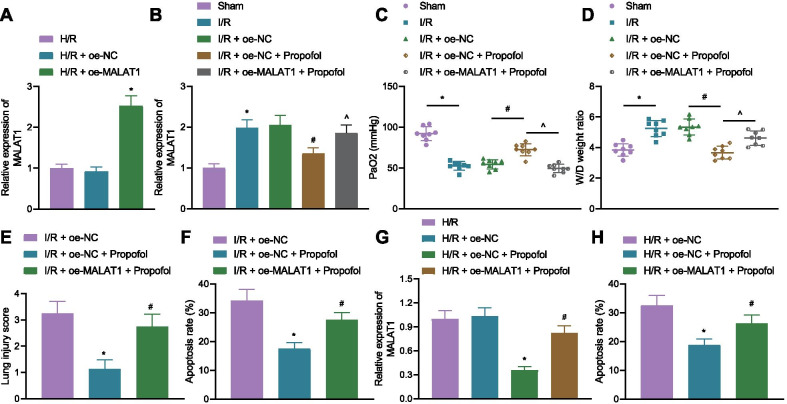
Overexpressing MALAT1 curtails alleviating effects of Propofol. **A** MALAT1 overexpression efficiency in cells as detected by RT-qPCR (*p < 0.05, compared with the oe-NC group). **B** MALAT1 overexpression efficiency in mice as detected by RT-qPCR (8 mice in each group; *p < 0.05, compared with that of the sham group; ^#^*p* < 0.05, compared with the I/R + oe-NC group; $ *p* < 0.05 compared with the I/R + oe-NC + Propofol group). **C** PaO_2_ level in mice (8 mice in each group; *p < 0.05, compared with that of the sham group; ^#^*p* < 0.05, compared with the I/R + oe-NC group; ^$^*p* < 0.05 compared with the I/R + oe-NC + Propofol group). **D** W/D ratio in lung tissues of mice (8 mice in each group; *p < 0.05, compared with that of the sham group; ^#^*p* < 0.05, compared with the I/R + oe-NC group; ^$^*p* < 0.05 compared with the I/R + oe-NC + Propofol group). **E** Pathological changes of lung tissues of mice as detected by H&E staining (8 mice each group; *p < 0.05, compared with that of the I/R + oe-NC group; ^#^*p* < 0.05, compared with the I/R + oe-NC + Propofol group). **F** Cell apoptosis in lung tissues as determined by TUNEL staining (8 mice each group; *p < 0.05, compared with that of the I/R + oe-NC group; ^#^*p* < 0.05, compared with the I/R + oe-NC + Propofol group). **G** Expression of MALAT1 in cells as measured by RT-qPCR (*p < 0.05, compared with that of the H/R + oe-NC group; ^#^*p* < 0.05, compared with the H/R + oe-NC + Propofol group). **H** Cell apoptosis as determined by flow cytometry (*p* < 0.05, compared with that of the H/R + oe-NC group; ^#^*p* < 0.05, compared with the H/R + oe-NC + Propofol group). Cellular experiments were repeated 3 times independently

Furthermore, to analyze the effects of MALAT1 on I/R injury, MALAT1 was overexpressed/silenced in I/R mice and H/R cells. RT-qPCR confirmed the MALAT1 overexpression/silence efficiency in cells and mice, as shown in Fig. 2A, B and Additional file 6: Fig. S3A, B.

Measurement on PaO_2_ and W/D ratio in mice revealed that compared with the mice in the I/R + oe-NC group, PaO_2_ was increased while W/D ratio was decreased in the mice of the I/R + oe-NC + Propofol group. Yet, overexpressed MALAT1 reduced PaO_2_ and accumulated W/D ratio (Fig. 2C, D). An opposite outcome was observed in I/R mice treated with sh-MALAT1 (Additional file 6: Fig. 3C, D). H&E and TUENL staining results revealed that lung tissue injury scored lower, and cell apoptosis in the lung tissues was weakened in the mice of the I/R + oe-NC + Propofol group. However, lung tissue injury scored higher, and cell apoptosis in the lung tissues was elevated after overexpressing MALAT1 (Fig. 2E, F). As expected, delivery of sh-MALAT1 led to opposite results (Additional file 6: Fig. 3E, F).

**Fig. 3:**
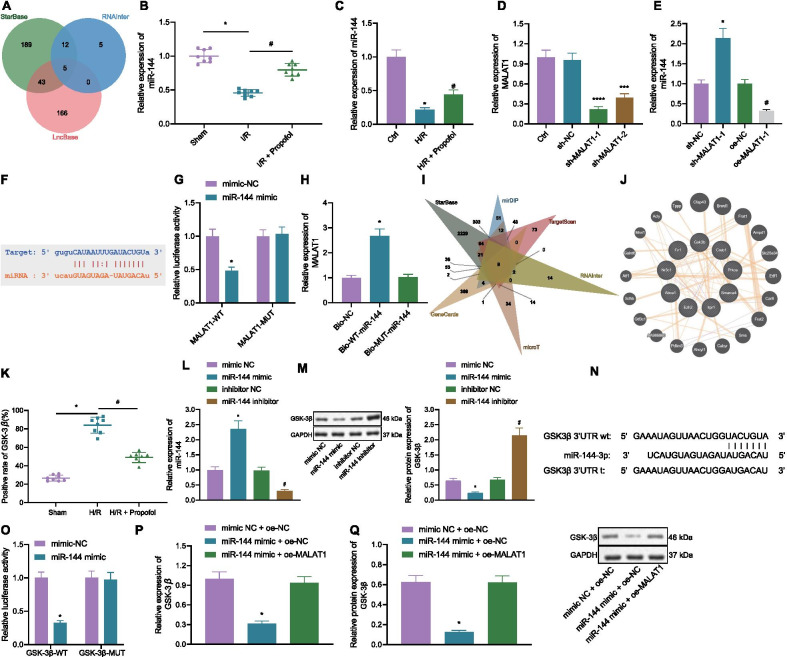
MALAT1 inhibited the expression of miR-144 to increase the expression of GSK3β. **A** Venn diagram showing the intersection of StarBase (http://starbase.sysu.edu.cn/), RNAInter (http://www.rna-society.org/rnainter/, Score ≥ 0.99), LncBase (http://carolina.imis.athena-innovation.gr/diana_Tools/web/Index.php?R=lncbasev2%2Findex, Score ≥ 0.9) results using the jvenn online tool (http://jvenn.toulouse.inra.fr/app/example.html). **B** Expression of miR-144 in lung tissues of mice as detected by RT-qPCR (8 mice in each group; *p < 0.05, compared with that of the sham group; ^#^*p* < 0.05, compared with the I/R group). **C** Expression of miR-144 in cells as detected by RT-qPCR (*p < 0.05, compared with the control group; ^#^*p* < 0.05, compared with the H/R group). **D** MALAT1 silencing efficiency as determined by RT-qPCR (***p = 0.0001, compared with the sh-NC group; ****p < 0.0001, compared with the sh-NC group). **E** Expression of miR-144 in cells as determined by RT-qPCR (*p < 0.05, compared with the sh-NC group; ^#^*p* < 0.05, compared with the oe-NC group). **F** Binding sites between MALAT1 and miR-144 as predicted by StarBase. **G** Binding relationship between MALAT1 and miR-144 as verified by dual-luciferase reporter gene assay (*p < 0.05, compared with mimic NC). **H** Binding of MALAT1 to miR-144 as measured by RNA pull-down (*p < 0.05, compared with Bio-NC). **I** Venn diagram showing the intersection of StarBase, mirDIP (http://ophid.utoronto.ca/mirDIP/, Score > 0.5), TargetScan (http://www.targetscan.org/vert_72/), RNAInter (http://www.rna-society.org/rnainter/, Score > 0.6) and microT (http://diana.imis.athena-innovation.gr/DianaTools/index.php?R=microT_CDS/, Score > 0.9) results and I/R-related genes. **J** Co-expression network as plotted using GeneMANIA tool (http://genemania.org/). **K** Expression of GSK3β in mice as assessed by immunohistochemistry (8 mice each group; *p < 0.05, compared with that of the sham group; ^#^*p* < 0.05, compared with the I/R group). **L** miR-144 overexpression and silencing efficiencies (*p < 0.05, compared with mimic NC; ^#^*p* < 0.05, compared with inhibitor NC). **M** Expression of GSK3β in cells as assessed by Western blotting (*p < 0.05, compared with mimic NC; ^#^*p* < 0.05, compared with inhibitor NC). **N** Binding sites and mutant sites between miR-144 and GSK3β. **O** Binding relationship between miR-144 and GSK3β as verified by dual-luciferase reporter gene assay (*p < 0.05, compared with mimic NC). **P** GSK3β expression in cells as determined by RT-qPCR (*p < 0.05, compared with mimic NC). **Q** GSK3β expression in cells as determined by Western blotting (*p < 0.05, compared with mimic NC + oe-NC). Cellular experiments were repeated 3 times independently

MALAT1 was downregulated in cells of the H/R + oe-NC + Propofol group, but upregulated after overexpressing MALAT1 (Fig. 2G). Also, apoptosis of cells in the H/R + oe-NC + Propofol group was diminished, yet overexpressing MALAT1 increased the rate of cell apoptosis (Fig. 2H). However, the presence of sh-MALAT1 in H/R cells significantly reduced the rate of cell apoptosis (Additional file 6: Fig. 3G). These results indicated that Propofol could downregulate MALAT1 to alleviate lung injury in I/R mice and H/R cells. However, overexpressed MALAT1 reversed the alleviating effects of Propofol.

### MALAT1 competitively binds to miR-144 and elevates GSK3β expression

To further explore the regulatory mechanism of MALAT1, bioinformatics analysis was performed through StarBase, RNAInter, and LncBase online tools, obtaining 249, 22, and 214 miRNAs as downstream of MALAT1, respectively. After intersection of the miRNAs, there were 5 candidate miRNAs, hsa-miR-140-5p, hsa-miR-144-3p (Previous IDs: hsa-miR-144), hsa-miR-101-3p, hsa-miR-1-3p, and hsa-miR-206 (Fig. 3A). Since the role of miR-144 was preliminarily studied in myocardial I/R, we further explored the impacts of miR-144 on lung I/R injury. RT-qPCR results showed that miR-144 was poorly expressed in I/R mice and H/R cells compared with that of the sham-operated mice and the control cells, respectively. When compared with the I/R mice and H/R cells, miR-144 expression was increased in mice of the I/R + Propofol group and the cells in the H/R + Propofol group (Fig. 3B, C). MALAT1 was further silenced, in which sh-MALAT1-1 was chosen for further assays (Fig. 3D). It was revealed that the expression of miR-144 was elevated after sh-MALAT1 treatment, yet oe-MALAT1 treatment reduced the expression of miR-144 (Fig. 3E). These results suggested that MALAT1 could inhibit the expression of miR-144.

The binding sites between MALAT1 and miR-144 were predicted by StarBase (Fig. 3F), and miRBase revealed that the binding sites showed conservation in human and mice. Dual-luciferase reporter gene assay verified that miR-144 mimic treatment strengthened the luciferase activity of MALAT1 WT, but had no significant change on that of MALAT1 MUT (Fig. 3G). RNA pull-down results displayed a more obvious binding interaction of Bio-WT-miR-144 to MALAT1 (Fig. 3H).

Furthermore, I/R mice and H/R cells were treated with miR-144 agomir and mimic, respectively. RT-qPCR for overexpression efficiency of miR-144 verified that miR-144 mimic/agomir resulted in high expression of miR-144 (Additional file 7: Fig. 4A, B). In addition, upregulated levels of miR-144 significantly increased PaO_2_ levels, lowered W/D ratio and lung tissue injury scores as well as reduced cell apoptosis rate in lung tissues (Additional file 7: Fig. 4C–F). Flow cytometric data revealed lower cell apoptosis rate in cells overexpressing miR-144 (Additional file 7: Fig. 4G).

**Fig. 4:**
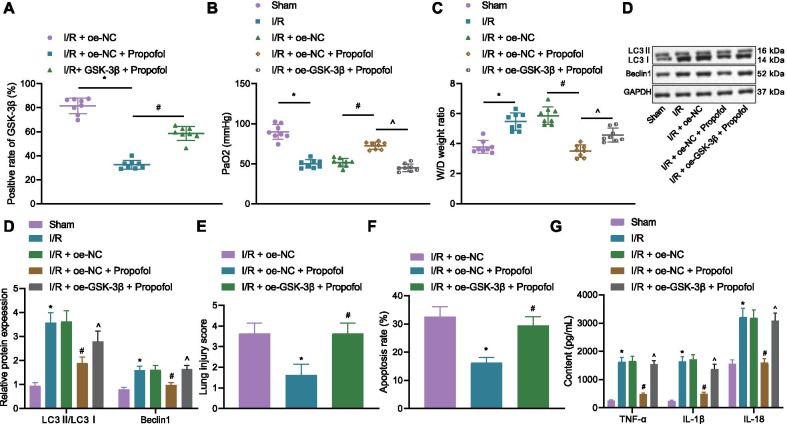
Overexpressing GSK3β facilitates autophagy and inflammation in I/R mice. **A** Expression of GSK3β in lung tissues of mice as determined by immunohistochemistry (8 mice in each group; *p < 0.05, compared with the I/R + oe-NC group; ^#^*p* < 0.05, compared with the I/R + oe-NC + Propofol group). **B** PaO_2_ level of mice (8 mice in each group; *p < 0.05, compared with the sham group; ^#^*p* < 0.05, compared with the I/R + oe-NC group; ^$^*p* < 0.05, compared with the I/R + oe-NC + Propofol group). **C** W/D ratio of mice (8 mice in each group; *p < 0.05, compared with the sham group; ^#^*p* < 0.05, compared with the I/R + oe-NC group; ^$^*p* < 0.05, compared with the I/R + oe-NC + Propofol group). **D** The expressions of GSK3β and autophagy-related protein levels in lung tissues as detected by Western blot analysis (8 mice in each group; *p < 0.05, compared with that of the sham group; ^#^*p* < 0.05, compared with the I/R + oe-NC group; ^$^*p* < 0.05, compared with the I/R + oe-NC + Propofol group). **E** Pathological changes of lung tissues of mice as detected by H&E staining (8 mice each group; *p < 0.05, compared with that of the I/R + oe-NC group; ^#^*p* < 0.05, compared with the I/R + oe-NC + Propofol group). **F** Cell apoptosis in lung tissues as determined by TUNEL staining (8 mice each group; *p < 0.05, compared with that of the I/R + oe-NC group; ^#^*p* < 0.05, compared with the I/R + oe-NC + Propofol group). **G** Levels of TNF-α, IL-1β, and IL-18 in supernatant of lung tissues of mice as measured by ELISA (8 mice in each group; *p < 0.05, compared with the sham group; ^#^*p* < 0.05, compared with the I/R + oe-NC group; ^$^*p* < 0.05, compared with the I/R + oe-NC + Propofol group). The experiment was repeated 3 times independently

Target genes of miR-144 were subsequently explored. StarBase, mirDIP, TargetScan, RNAInter, and microT online databases predicted that there were 3,655, 1,388, 1,048, 695, and 535 mRNAs as downstream mRNAs of miR-144, respectively. mRNAs were intersected with the results from GeneCards, obtaining the following 9 candidate target genes, SMARCA4, FN1PRKCE, CREB1, GSK3B, ITPR1, NR3C1, EZH2, and ABCA1 (Fig. 3I). Co-expression network was plotted using GeneMANIA (Fig. 3J), in which GSK3B (Alias: GSK3β) was the hub gene (Additional file 8: Table S4). Immunohistochemistry results showed that the expression of GSK3β was elevated in I/R mice, but reduced in mice of the I/R + Propofol group (Fig. 3K). Additionally, miR-144 mimic treatment increased the expression of miR-144 and reduced the expression of GSK3β, while miR-144 inhibitor decreased the expression of miR-144 and elevated the expression of GSK3β (Fig. 3L, M). The results indicated that miR-144 may suppress GSK3β expression.

Furthermore, bioinformatics analysis predicted that miR-144 targeted the binding site of GSK3β 3’UTR (Fig. 3N) region, and dual-luciferase reporter gene assay verified that miR-144 targeted GSK3β and inhibited its expression (Fig. 3O). GSK3β was downregulated in the miR-144 mimic + oe-NC group compared with that of the mimic NC + oe-NC group, while GSK3β was upregulated in the miR-144 mimic + oe-MALAT1 group compared with that of the miR-144 mimic + oe-NC group (Fig. 3P, Q). These results indicated that MALAT1 bound to miR-144 to upregulate GSK3β.

### Overexpression of GSK3β reverses protective effects of propofol on lung injury in I/R mouse

The relation between Propofol and GSK3β in I/R mice was further studied. The expression of GSK3β was increased in I/R mice compared with that in the sham-operated mice. The expression of GSK3β was decreased in mice of the I/R + oe-NC + Propofol group when compared with that of the mice in the I/R + oe-NC group. However, GSK3β expression was further increased in the mice of the I/R + oe-GSK3β + Propofol group relative to that of the I/R + oe-NC + Propofol group (Fig. 4A). PaO_2_ was reduced but W/D ratio was increased in I/R mice relative to that of sham-operated mice, while PaO_2_ was increased but W/D ratio was reduced in mice of the I/R + oe-NC + Propofol group when compared with those in the I/R + oe-NC group; compared with the I/R + oe-NC + Propofol group, mice in the I/R + oe-GSK3β + Propofol group showed decreased PaO_2_ levels and increased W/D ratios (Fig. 4B, C). Autophagy-related proteins in lung tissues of mice were determined by Western blot analysis. LC3II/LC3I ratio and the expression of Beclin1 were increased in I/R mice relative to that of sham-operated mice, while LC3II/LC3I ratio and the expression of Beclin1 were reduced in mice of the I/R + oe-NC + Propofol group when compared with those in the I/R + oe-NC group; compared with the I/R + oe-NC + Propofol group, mice in the I/R + oe-GSK3β + Propofol group showed elevated LC3II/LC3I ratio and expression of Beclin1 (Fig. 4D). Lung tissue injury scores and rate of cell apoptosis were elevated in I/R mice relative to that in sham-operated mice, while lung tissue injury scores and rate of cell apoptosis were reduced in mice of the I/R + oe-NC + Propofol group when compared with those in the I/R + oe-NC group; compared with the I/R + oe-NC + Propofol group, mice in the I/R + oe-GSK3β + Propofol group showed increased lung tissue injury scores and rate of cell apoptosis (Fig. 4E-F). I/R mice presented with elevated inflammatory factors tumor necrosis factor-α (TNF-α), interleukin-1β (IL-1β), and IL-18 levels, but those were reduced in mice of the I/R + oe-NC + Propofol group. However, overexpressed GSK3β increased TNF-α, IL-1β, and IL-18 levels (Fig. 4G). These results indicated that overexpressed GSK3β promoted the activation of autophagy and release of inflammatory factors to reverse the protective effects of Propofol on I/R mice.

### Overexpression of GSK3β reverses protective effects of propofol on H/R PMVEC model

**Fig. 5:**
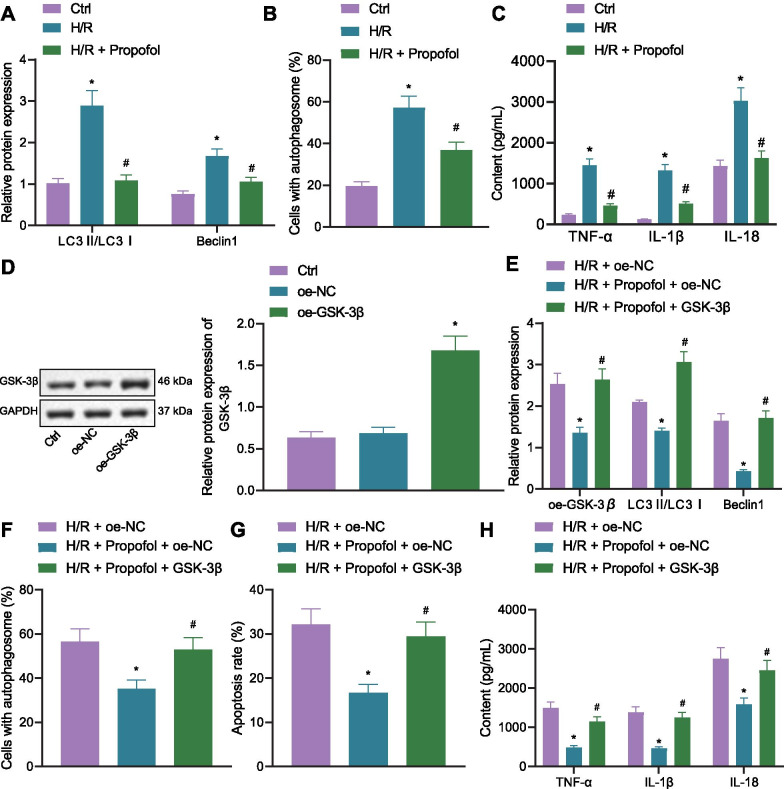
Overexpressed GSK3β inhibits protective effects of Propofol on H/R cells. **A** Autophagy-related protein expression in cells as detected by Western blot analysis (*p < 0.05, compared with that of the control group; ^#^*p* < 0.05, compared with the H/R group). **B** Autophagosome amount in cells as measured by immunofluorescence against LC3 (*p < 0.05, compared with that of the control group; ^#^*p* < 0.05, compared with the H/R group). **C** Inflammatory factor levels in cells as detected by ELISA (*p < 0.05, compared with the control group; ^#^*p* < 0.05, compared with the H/R group). **D** GSK3β overexpression efficiency as detected by Western blot analysis. E, Expression of GSK3β and autophagy-related protein levels as determined by Western blot analysis (*p < 0.05, compared with that of the H/R + oe-NC group; ^#^*p* < 0.05, compared with the H/R + oe-NC + Propofol group). **F** Autophagosome amount in cells as measured by immunofluorescence against LC3 (*p < 0.05, compared with the H/R + oe-NC group; ^#^*p* < 0.05, compared with the H/R + oe-NC + Propofol group). **G** Cell apoptosis as assessed by flow cytometry (*p < 0.05, compared with the H/R + oe-NC group; ^#^*p* < 0.05, compared with the H/R + oe-NC + Propofol group). H, Inflammatory factor levels as determined by ELISA (*p < 0.05, compared with the H/R + oe-NC group; ^#^*p* < 0.05, compared with the H/R + oe-NC + Propofol group). The experiment was repeated 3 times independently

Promotive effects of GSK3β on lung I/R injury were further explored. In H/R cells, LC3II/LC3I ratio and the expression of Beclin1 were increased, yet the addition of Propofol decreased the LC3II/LC3I ratio and the expression of Beclin1 (Fig. 5A). The number of autophagosomes was increased in H/R cells, but reduced after addition of Propofol (Fig. 5B). TNF-α, IL-1β, and IL-18 levels were increased in H/R cells, but also decreased after addition of Propofol (Fig. 5C). These results suggested that Propofol decreased the number of autophagosomes and release of inflammatory factors.

GSK3β was overexpressed and Western blot analysis results validated that the expression of GSK3β was increased after oe-GSK3β treatment (Fig. 5D). It was shown that GSK3β expression, LC3II/LC3I ratio and Beclin1 expression were declined in the cells of the H/R + oe-NC + Propofol group, along with decreased numbers of autophagosomes. The results were reversed by overexpressing GSK3β (Fig. 5E, F). Also, the rate of cell apoptosis was decreased in the cells of the H/R + oe-NC + Propofol group, but increased by overexpressing GSK3β (Fig. 5G). TNF-α, IL-1β, and IL-18 levels were reduced in the cells of the H/R + oe-NC + Propofol group, but elevated by overexpressing GSK3β (Fig. 5H). These results indicated that overexpressed GSK3β promoted the activation of autophagy and release of inflammatory factors to reverse the protective effects of Propofol on H/R cells.

### Propofol reduces autophagy activation and release of inflammatory factors via the MALAT1/miR-144/ GSK3β axis in lung tissues of I/R mice

**Fig. 6:**
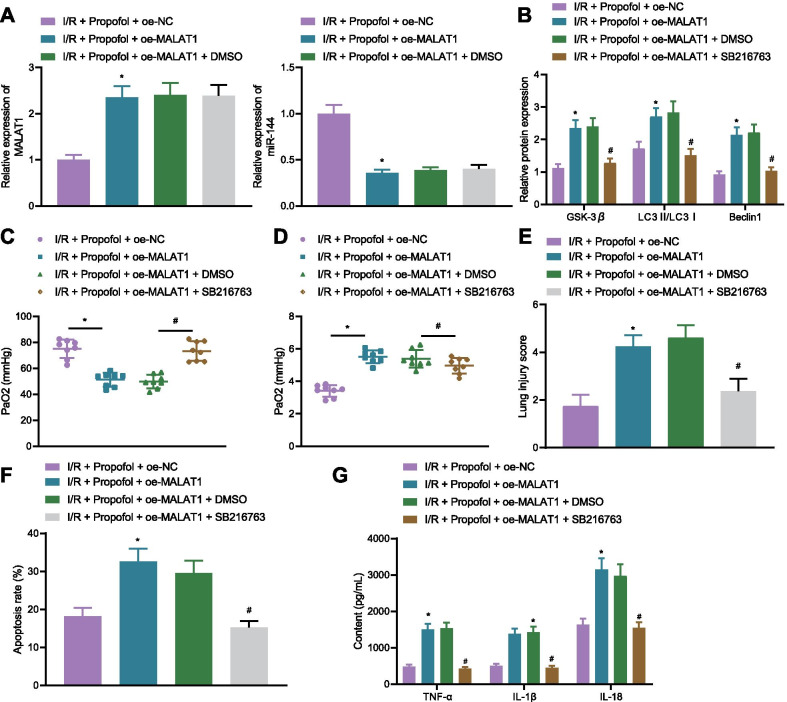
Propofol relieved lung I/R injury in mice through the MALAT1/miR-144/GSK3β axis. **A** Expressions of MALAT1 and miR-144 in mice as detected by RT-qPCR (8 mice in each group; *p < 0.05, compared with that of the I/R + oe-NC + Propofol group). **B** Autophagy-related protein levels as detected by Western blot analysis (8 mice in each group; *p < 0.05, compared with that of the I/R + oe-NC + Propofol group; ^#^*p* < 0.05, compared with I/R + Propofol + oe-MALAT1 + DMSO group). **C** PaO_2_ in mice (8 mice in each group; *p < 0.05, compared with the I/R + oe-NC + Propofol group; ^#^*p* < 0.05, compared with I/R + Propofol + oe-MALAT1 + DMSO group). **D** W/D ratio in mice (8 mice in each group; * *p* < 0.05, compared with the I/R + oe-NC + Propofol group; ^#^*p* < 0.05, compared with I/R + Propofol + oe-MALAT1 + DMSO group). **E** Pathological changes of lung tissues of mice as detected by H&E staining 8 mice each group; *p < 0.05, compared with that of the I/R + oe-NC + Propofol group; ^#^*p* < 0.05, compared with the I/R + Propofol + oe-MALAT1 + DMSO group). **F** Cell apoptosis in lung tissues as determined by TUNEL staining (8 mice each group; *p < 0.05, compared with that of the I/R + oe-NC + Propofol group; ^#^*p* < 0.05, compared with the I/R + Propofol + oe-MALAT1 + DMSO group). **G** Levels of TNF-α, IL-1β, and IL-18 in supernatant of lung tissues of mice as measured by ELISA (8 mice each group; *p < 0.05, compared with the I/R + oe-NC + Propofol group; ^#^*p* < 0.05, compared with the I/R + Propofol + oe-MALAT1 + DMSO group). The experiment was repeated 3 times independently

To verify if Propofol alleviated lung I/R injury through MALAT1/miR-144/GSK3β in vivo, GSK3β inhibitor, SB216763, were injected into mice. MALAT1 was highly expressed in mice of the I/R + oe-MALAT1 + Propofol group, while miR-144 was poorly expressed, along with increased expression of GSK3β, LC3II/LC3I ratio, and the expression of Beclin1 (Fig. 6A, B). However, after addition of SB216763, expression of GSK3β, LC3II/LC3I ratio, and the expression of Beclin1 were reduced (Fig. 6B). Meanwhile, PaO_2_ was declined while W/D ratio was accumulated in mice of the I/R + oe-MALAT1 + Propofol group, yet the addition of SB216763 increased PaO_2_ levels and decreased W/D ratio (Fig. 6C). H&E and TUNEL staining results revealed that lung tissue injury scores and rate of cell apoptosis in lung tissues of mice in the I/R + oe-MALAT1 + Propofol group were increased, but were reduced after injection of SB216763 (Fig. 6E, F). Levels of inflammatory factors, TNF-α, IL-1β, and IL-18 were elevated in mice of the I/R + oe-MALAT1 + Propofol group, and injection of SB216763 reduced the levels of TNF-α, IL-1β, and IL-18 (Fig. 6G). These results suggested that Propofol decreased the number of autophagosomes and release of inflammatory factors in the lung tissues of I/R mice via the MALAT1/miR-144/GSK3β axis.

### Propofol reduces autophagy activation and release of inflammatory factors via the MALAT1/miR-144/ GSK3β axis in H/R cell model

**Fig. 7:**
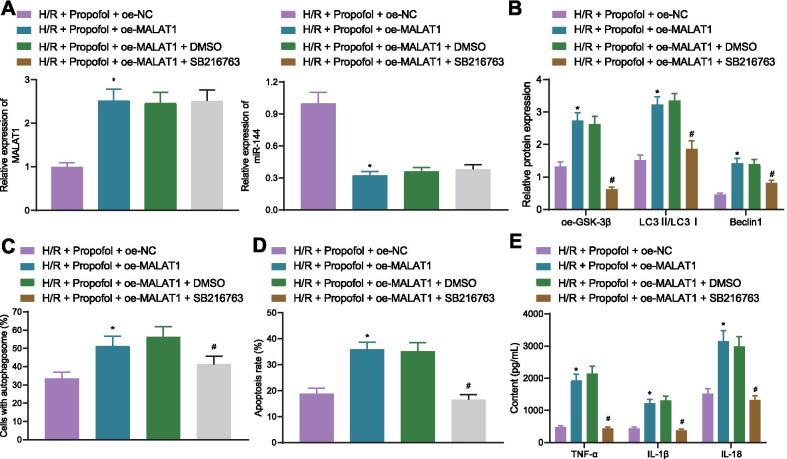
Propofol alleviates I/R injury through the MALAT1/miR-144/GSK3β axis in vitro*.* **A** Expressions of MALAT1 and miR-144 in cells as detected by RT-qPCR (*p < 0.05, compared with the H/R + oe-NC + Propofol group). **B** Autophagy-related protein levels as detected by Western blotting (*p < 0.05, compared with the H/R + oe-NC + Propofol group; ^#^*p* < 0.05, compared with H/R + Propofol + oe-MALAT1 + DMSO group). **C** Amount of autophagosome as determined by immunofluorescence against LC3 (*p < 0.05, compared with the H/R + oe-NC + Propofol group; ^#^*p* < 0.05, compared with the H/R + Propofol + oe-MALAT1 + DMSO group). **D** Cell apoptosis as determined by flow cytometry (*p < 0.05, compared with the H/R + oe-NC + Propofol group; ^#^*p* < 0.05, compared with the H/R + Propofol + oe-MALAT1 + DMSO group). **E** Levels of TNF-α, IL-1β, and IL-18 in supernatant of cells as measured by ELISA (*p < 0.05, compared with the H/R + oe-NC + Propofol group; ^#^*p* < 0.05, compared with the H/R + Propofol + oe-MALAT1 + DMSO group). Cellular experiments are repeated 3 times independently

Lastly, to further demonstrate that the contribution of Propofol to H/R injury alleviation by inhibiting cell autophagy and inflammation was dependent on the MALAT1/miR-144/GSK3β axis in vitro, cells were treated with GSK3β inhibitor SB216763. RT-qPCR results exhibited that the expression of MALAT1 was increased in the cells of the H/R + oe-MALAT1 + Propofol group, while miR-144 expression was reduced (Fig. 7A). Additionally, in the cells of the H/R + Propofol + oe-MALAT1 group, expression of GSK3β, LC3II/LC3I ratio, and the expression of Beclin1 were increased, which were decreased in cells of the H/R + Propofol + oe-MALAT1 + SB216763 group (Fig. 7B). Cells of the H/R + oe-MALAT1 + Propofol group also showed increased numbers of autophagosomes, rate of cell apoptosis, and inflammatory factor levels. However, in cells of the H/R + Propofol + oe-MALAT1 + SB216763 group, the number of autophagosomes, cell apoptosis, and inflammatory factor levels were reduced (Fig. 7C–E). These results suggested that Propofol suppressed autophagy activation and inflammatory factor release in H/R cells though the MALAT1/miR-144/GSK3β axis to relieve the injury of PMVECs induced by H/R.

## Discussion

**Fig. 8:**
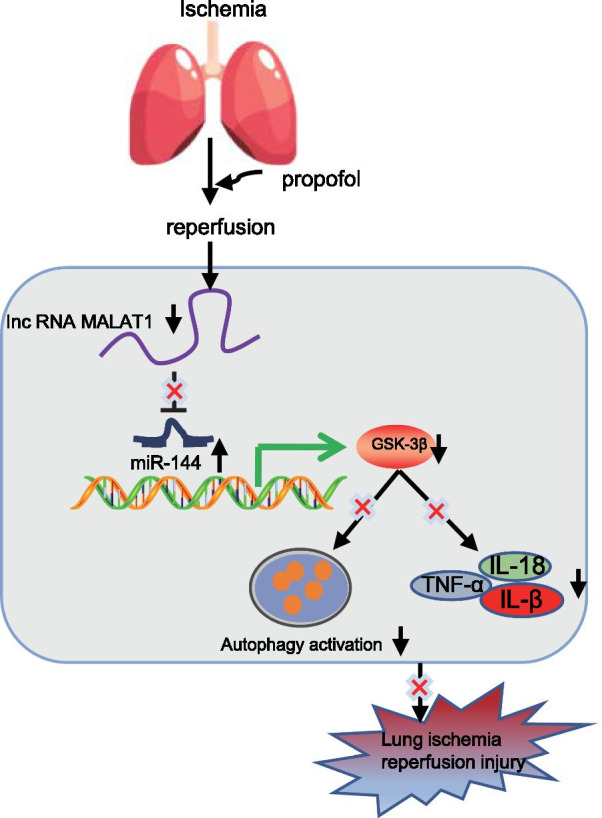
The molecular scheme showing the impacts of Propofol involving the MALAT1/miR-144/GSK3β axis on lung I/R injury

As lung I/R injury and its associated inflammatory responses are the mainstay obstacles for lung transplantation, it is urgent to find therapeutic targets for alleviating lung injury induced by I/R (Laubach and Sharma 2016). Accumulating studies have illustrated the protective effects of Propofol on lung diseases, such as lung epithelial injury induced by I/R and oxidative stress (Balyasnikova et al. 2005; Yang et al. 2014). However, our understanding on its underlying mediators and mechanisms is still limited. In the present study, we established an I/R injury mouse model as well as an H/R cell model to investigate the effect of Propofol on I/R-induced lung injury with the involvement of the MALAT1/miR-144/GSK3β axis. We concluded that Propofol could upregulate the expression of miR-144 by suppressing the expressions of MALAT1 and GSK3β, thus inhibiting the activation of autophagy and inflammation in lung I/R injury (Fig. 8).

Our research confirmed that in I/R mouse models and H/R cell models, a significant high expression of MALAT1 was detected. MALAT1 has been found to be highly expressed in lung transplant I/R, and silenced MALAT1 plays an alleviating role in inflammatory injury after lung transplant I/R (Wei et al. 2019). Likewise, upregulated levels of MALAT1 have been identified in lung tissues of mice with septic lung injury while MALAT1 knockdown significantly improves lipopolysaccharide-induced pathological injury of lungs (Lin et al. 2019). However, after treatment of Propofol, the expression of MALAT1 was evidently reduced, which indicated that Propofol may downregulate the expression of MALAT1. Propofol reducing the expression of MALAT1 has also been demonstrated in gastric cancer, in which Propofol decreased the expression of MALAT1 to promote the sensitivity of gastric cancer cells to cisplatin (Zhang et al. 2020). Furthermore, the experimental results revealed that overexpressing MALAT1 could reverse the protective effects of Propofol on lung I/R injury, which has been widely reported in various contexts (Ruan et al. 2020; Huang et al. 2019; Yu and Li 2019). As previously proposed, MALAT1 represents a diagnostic target for chronic obstructive pulmonary disease (Hu et al. 2020). These findings supported our statement that MALAT1 was highly expressed in I/R injury, and that Propofol could prevent lung I/R injury by reducing the expression of MALAT1 in I/R mice and H/R cells.

Our further mechanistic study revealed that the MALAT1/miR-144/GSK3β axis played an important role in I/R mice and H/R cells. During the process of lung I/R injury, MALAT1 was highly expressed, which targeted miR-144. Previous references have reported that MALAT1 could target miR-144, and negatively regulate the expression of miR-144 after myocardial infarction (Gong et al. 2019). In addition, a prior study indicated that miR-144 could block the endothelial cell barrier as well as hyperpermeability in lungs to restrain inflammatory response (Siddiqui et al. 2019). Moreover, miR-144 is downregulated by I/R injury, and plays a cardio-protective role in ischemic preconditioning accompanied by elevated extent of GSK3β phosphorylation in the myocardium (Li et al. 2014). Our bioinformatics analysis results suggested that GSK3β was a hub gene which may be regulated by miR-144. GSK3β has been reported to aggravate lung I/R injury while inhibiting GSK3β mends lung function following I/R injury (Waldow et al. 2010). It has been reported that GSK3β is a pro-inflammatory agent, and that inhibiting the expression of GSK3β inhibits the inflammatory response and can be achieved by inhibiting autophagy to suppress NLRP3 inflammasome activation, therefore alleviating cerebral I/R injury in rats (Tantray et al. 2017; Wang et al. 2019). The validation of our results on the relation between miR-144 and GSK3β requires further investigations in the future.

To confirm whether Propofol affected the MALAT1/miR-144/GSK3β axis during lung I/R injury, we tested the lung tissues of mice and PMVECs in different treatment groups. Our results concluded that the injection of Propofol relieved I/R injury by increasing PaO_2_ levels and decreasing W/D ratio. A previous study stated that I/R injury could increase W/D ratio while decreasing PaO_2_ level (Zou and Sun 2020). PaO_2_ can be significantly improved by the pretreatment of Propofol along with reduced lung W/D weight ratio in lung tissues of rats with oleic acid-induced acute lung injury (Tan et al. 2018). Therefore, it was reasonable to conclude that Propofol alleviated I/R in vivo*.* Furthermore, Propofol treatment reduced LC3II/LC3I ratio, expressions of Beclin1, and number of autophagosomes. The prevention of autophagy and apoptosis are correlated with lower ratios of LC3II/LC3I and reduced expression of Beclin1 (He et al. 2016). We also found that Propofol reduced levels of TNF-α, IL-1β, and IL-18. I/R injury is accompanied by the increase of pro-inflammatory factors TNF-α, IL-1β, and IL-18 (Matsuo et al. 2013). The important role of Propofol in autophagy has been widely reported (Guo and Ma 2020). Specifically, Propofol is capable of repressing renal I/R-induced pulmonary autophagy and apoptosis (Liu et al. 2020). These findings proved that Propofol alleviated lung I/R injury by inactivating autophagy and restraining inflammation.

Although Propofol has been verified to inhibit inflammatory responses by regulating the MALAT1/miR-144/GSK3β axis during lung I/R injury, we have not conducted extensive research on other potentially related mechanisms, thus warranting further exploration. In conclusion, Propofol can upregulate the expression of miR-144 by reducing the expressions of MALAT1 and GSK3β, hence inhibiting the activation of autophagy and inflammation in lung injury caused by I/R.

## Conclusions

We preliminary drew the conclusion that Propofol downregulated MALAT1 to promote the suppression of miR-144 on GSK3β, which inactivated autophagy and suppressed the release of inflammatory factors, eventually alleviating lung I/R injury (Fig. 8). This study may provide new therapeutic targets for the prevention of lung I/R injury.

## Supplementary Information

**Additional file 1: Fig. S1.** Schematic diagram of lung I/R injury model establishment.

**Additional file 2: Table S1. **The sequences for silencing lentivirus

**Additional file 3: Fig. S2.** Schematic diagram showing the establishment of H/R PMVEC model

**Additional file 4: Table S2. **Primer sequences for RT-qPCR.

**Additional file 5: Table S3. **I/R injury related lncRNAs.

**Additional file 6: Fig. S3.** Silencing MALAT1 alleviates lung I/R injury. **A** MALAT1 silence efficiency in cells as detected by RT-qPCR (*p < 0.05, compared with the H/R + sh-NC group). **B** MALAT1 silence efficiency in mice as detected by RT-qPCR (8 mice in each group; *p < 0.05, compared with the I/R + sh-NC group). **C** PaO_2_ level in mice (8 mice in each group; *p < 0.05, compared with the I/R + sh-NC group). **D** W/D ratio in lung tissues of mice (8 mice in each group; *p < 0.05, compared with the I/R + sh-NC group). **E** Pathological changes of lung tissues of mice as detected by H&E staining (scale bar: 20 μm, 8 mice each group; * *p* < 0.05, compared with the I/R + sh-NC group). **F** Cell apoptosis in lung tissues as determined by TUNEL staining (scale bar: 50 μm, 8 mice each group; *p < 0.05, compared with the I/R + sh-NC group). **G** Cell apoptosis as determined by flow cytometry (*p* < 0.05, compared with the H/R + sh-NC group). The experiment was repeated 3 times independently.

**Additional file 7: Fig. S4.** Upregulating miR-144 alleviates lung I/R injury. **A** Expression of miR-144 in cells as detected by RT-qPCR (*p < 0.05, compared with the H/R + mimic-NC group). **B** Expression of miR-144 in lung tissues of mice as detected by RT-qPCR (8 mice in each group; *p < 0.05, compared with that of the I/R + agomir-NC group). **C** PaO_2_ level in mice (8 mice in each group; * *p* < 0.05, compared with the I/R + agomir-NC group). **D** W/D ratio in lung tissues of mice (8 mice in each group; *p < 0.05, compared with the I/R + agomir-NC group). **E** Pathological changes of lung tissues of mice as detected by H&E staining (scale bar: 20 μm, 8 mice each group; *p < 0.05, compared with the I/R + agomir-NC group). **F** Cell apoptosis in lung tissues as determined by TUNEL staining (scale bar: 50 μm, 8 mice each group; *p < 0.05, compared with the I/R + agomir-NC group). **G** Cell apoptosis as determined by flow cytometry (*p* < 0.05, compared with the H/R + mimic-NC group). The experiment was repeated 3 times independently.

**Additional file 8: Table S4**. The key target genes of miR-144 in lung I/R injury selected according to the co-expression score.

## Data Availability

The datasets generated/analysed during the current study are available.

## Abbreviations

I/R: Ischemia/reperfusion
lncRNAs: Long non-coding RNAs
MALAT1: Metastasis-associated lung adenocarcinoma transcript 1
GSK3β: Glycogen synthase kinase-3β
H/R: Hypoxia/reperfusion
H&E: Hematoxylin & eosin
PBS: Phosphate buffer saline
HRP: Horseradish peroxide
EDTA: Ethylene diamine tetraacetic acid
DMEM: Dulbecco’s modified eagle medium
cck: Cell counting kit
FITC: Fluorescein isothiocyanate
UTR: Untranslated region
WT: Wild type
MUT: Mutant type
RIPA: Radio-immunoprecipitation assay
BCA: Bicinchoninic acid
PVDF: Polyvinylidene fluoride
TBST: Tris-buffered saline Tween
ECL: Enhanced chemiluminescence
