# Supplementary material for: Propofol attenuates lung ischemia/reperfusion injury though the involvement of the MALAT1/microRNA-144/GSK3β axis
Source: Mol Med. 2021 Jul 15;27:77. doi: 10.1186/s10020-021-00332-0 (PMC8281462; doi:10.1186/s10020-021-00332-0)
Supplement: Supplementary file 2 — Additional file 2: Table S1. The sequences for silencing lentivirus [file 10020_2021_332_MOESM2_ESM.docx]

**Supplementary Table 1** The sequences for silencing lentivirus

| Silencing lentivirus | Silencing sequences (5'-3') |
| --- | --- |
| sh-NC | GACCTGTACGCCAACACAGTG |
| sh-MALAT1-1 (sh-MALAT1) | TCCACTTGATCCCAACTCATC |
| sh-MALAT1-2 | TTCCTTAGTTGGCATCAAGGC |

Notes: NC, negative control; MALAT, metastasis-associated lung adenocarcinoma transcript; sh, short hairpin RNA.
