# Supplementary material for: Propofol attenuates lung ischemia/reperfusion injury though the involvement of the MALAT1/microRNA-144/GSK3β axis
Source: Mol Med. 2021 Jul 15;27:77. doi: 10.1186/s10020-021-00332-0 (PMC8281462; doi:10.1186/s10020-021-00332-0)
Supplement: Supplementary file 4 — Additional file 4: Table S2. Primer sequences for RT-qPCR. [file 10020_2021_332_MOESM4_ESM.docx]

**Supplementary Table 2** Primer sequences for RT-qPCR

|  | Primer sequences (5'-3') |
| --- | --- |
| MALAT1 | F: AAAGCAAGGTCTCCCCACAAG |
|  | R: GGTCTGTGCTAGATCAAAAGGCA |
| miR-144 | F: CCTCGCACCTGGAGGCTGGCTG |
|  | R: TTATCAGTTGGGAAAATAGTTA |
| U6 | F: TCGTCGCCCACATAGGAATC |
|  | R: CGCTTCGGCAGCACATATAC |
| GAPDH | F: CAGTGCCAGCCTCGTCTCAT |
|  | R: AGGGGCCATCCACAGTCTTC |

Notes: RT-qPCR, reverse transcription quantitative polymerase chain reaction; MALAT1, metastasis-associated lung adenocarcinoma transcript; miR-144, microRNA-144; GAPDH, glyceraldehye phosphate dehydrogenase; F, forward; R, reverse.
