# Supplementary material for: Propofol attenuates lung ischemia/reperfusion injury though the involvement of the MALAT1/microRNA-144/GSK3β axis
Source: Mol Med. 2021 Jul 15;27:77. doi: 10.1186/s10020-021-00332-0 (PMC8281462; doi:10.1186/s10020-021-00332-0)
Supplement: Supplementary file 5 — Additional file 5: Table S3. I/R injury related lncRNAs. [file 10020_2021_332_MOESM5_ESM.docx]

**Supplementary Table 3** I/R injury related lncRNAs

| ncRNA Symbol | ncRNA Category | Species | Disease Name | Score |
| --- | --- | --- | --- | --- |
| hsa-miR-136-5p | miRNA | Homo sapiens | Ischemia/reperfusion injury | 1 |
| lncRNA-N1LR | lncRNA | Mus musculus | Ischemia/reperfusion injury | 1 |
| Malat1 | lncRNA | Mus musculus | Ischemia/reperfusion injury | 0.99998 |
| rno-mir-199a | miRNA | Rattus norvegicus | Ischemia/reperfusion injury | 0.973352 |
| rno-mir-21 | miRNA | Rattus norvegicus | Ischemia/reperfusion injury | 0.973352 |
| rno-mir-24 | miRNA | Rattus norvegicus | Ischemia/reperfusion injury | 0.973352 |
